# Autologous Pericardial Cusps Within a Tubularized Bovine Pericardial Conduit for Right Ventricular Outflow Tract Reconstruction During the Modified Ross Procedure: A Technical Note

**DOI:** 10.3390/jcm15197684

**Published:** 2026-10-04

**Authors:** Tomáš Toporcer, Lukáš Vajda, Anton Bereš, Jaroslav Benedík, Marián Homola, Michal Trebišovský, Peter Šafár, Štefan Lukačín, Vilém Rohn, Adrián Kolesár

**Affiliations:** 1 Department of Heart Surgery, East Slovak Institute of Cardiovascular Diseases and Medical Faculty, Pavol Jozef Šafárik University, 04011 Košice, Slovakia; topyto@gmail.com (T.T.); lvajda@vusch.sk (L.V.); aberes@vusch.sk (A.B.); mtrebisovsky@vusch.sk (M.T.); psafar@vusch.sk (P.Š.); slukacin@vusch.sk (Š.L.); akolesar@vusch.sk (A.K.); 2 Department of Cardiac Surgery, Na Homolce Hospital, 15030 Prague, Czech Republic; jaroslav.benedik@uk-essen.de; 3 I. Surgery Department—Cardiovascular Surgery, General University Hospital and Second Faculty of Medicine, Charles University, 12808 Prague, Czech Republic; vilem.rohn@lf1.cuni.cz

**Keywords:** valved pericardial conduit, right ventricular outflow tract reconstruction, modified Ross procedure, autologous pericardium, Ozaki technique

## Abstract

**Background/Objectives:** Limited availability of pulmonary homografts may restrict right ventricular outflow tract reconstruction after the Ross procedure. This technical note describes the geometric construction of a valved conduit composed of autologous pericardial cusps and a tubularized bovine pericardial patch. **Methods:** Autologous pericardium (approximately 10 × 8 cm) is treated with 0.9% glutaraldehyde for 10 min, and three size-4 cusps with triangular ears are fashioned using a Fehling template. A bovine pericardial patch (14 × 9 cm) is marked with the cusp attachment baseline, the commissural fixation line, and asymmetric tubularization lines. The cusp implantation field is divided into three equal segments, and each cusp is sutured to the mesothelial surface of the patch with a continuous suture, followed by commissural fixation. The patch is then tubularized, and valve competence is assessed by saline testing. **Results:** The conduit was constructed in approximately 60 min, in parallel with the main operation, and implanted in four patients undergoing a modified Ross procedure in 2025. Echocardiography on the first postoperative day showed no more than trace pulmonary regurgitation and no conduit obstruction. No conduit-related complications occurred; only early postoperative follow-up is available. **Conclusions:** The technique allows construction of a valved conduit from autologous and bovine pericardium using widely available materials. Currently, it demonstrates technical feasibility only; hemodynamic performance, safety, and durability remain to be established in larger studies with standardized follow-up.

## 1. Introduction

Aortic valve stenosis is the most common valvular heart disease requiring intervention in developed countries [1]. Given the suboptimal properties of both mechanical and biological valve prostheses, aortic root replacement using a pulmonary autograft, as performed in the Ross procedure, remains a valuable surgical option, particularly in appropriately selected younger patients. However, the classical Ross procedure depends on the availability of pulmonary homografts, which varies among centers and has prompted the search for alternative options for pulmonary valve and right ventricular outflow tract reconstruction [2]. Although pulmonary homografts are regarded as the best available solution, stentless xenografts and other bioprosthetic conduits are used as alternatives, with an inferior expected durability [3]. In addition, there is a separate group of patients with pulmonary valve or right ventricular outflow tract disease in whom surgical replacement or reconstruction is indicated, even in centers without an established Ross program.

The aim of this study was to present a technical approach for the creation of a valved conduit for pulmonary valve and right ventricular outflow tract reconstruction. The conduit combines autologous pericardial cusps with a tubularized bovine pericardial patch and may be applicable in any patient with sufficient intact pericardium, that is, in patients without pericarditis, extensive pericardial resection, or previous cardiac surgery.

## 2. Technical Description

### 2.1. Harvesting and Treatment of Autologous Pericardium

**Figure 1:**
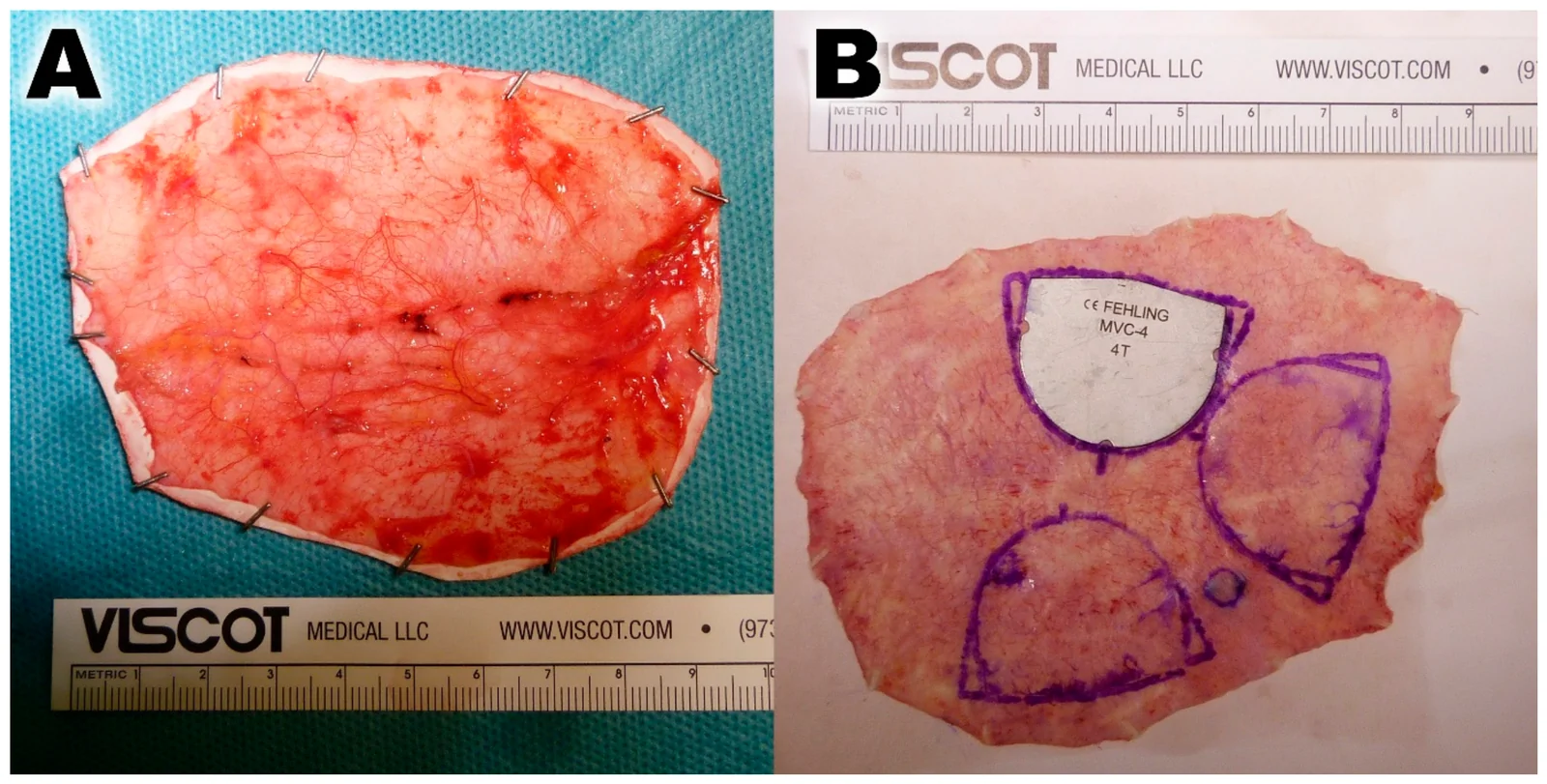
Intraoperative photograph of the pericardium. (**A**) Fresh autologous pericardium after harvesting. (**B**) The same pericardium after glutaraldehyde treatment, with one site of tissue damage indicated. Three valve cusps are outlined before excision according to the Fehling template (size 4). The superior portion of each cusp is extended by triangular “ears” measuring approximately 2.5 × 5 mm.

For conduit construction, an autologous pericardial patch measuring approximately 10 × 8 cm is required. This size is sufficient to prepare four valve cusps of size 4 using the Fehling template. The pericardium is harvested from the anterior portion of the pericardial sac, taking care to avoid injury to the phrenic nerve. The resulting pericardial defect is left open, as after routine coronary surgery. After harvesting, further preparation may be performed on a separate side table outside the operative field, in parallel with the main surgical procedure. The pericardial patch is secured along its edges to a firm, non-adherent surface using metal clips (Figure 1A). A sterile plastic sheet or sterile waxed paper may be used as the underlying surface. The mesothelial surface, corresponding to the pericardial cavity, is placed against this surface. The pericardium is treated with 0.9% glutaraldehyde solution (80 mL prepared from 25% glutaraldehyde solution (25% in water; Acros Organics, cat. no. 119980010, Thermo Fisher Scientific, Waltham, Massachusetts, USA) and 0.9% sodium chloride irrigation solution (Versylene^®^ Fresenius, Fresenius Kabi AG, Bad Homburg, Germany) in the appropriate proportion to achieve the desired final glutaraldehyde concentration) for 10 min at room temperature (approximately 22 °C), followed by two rinses in physiological saline (0.9% NaCl; 80 mL each), each lasting 5 min. Mechanical removal of residual fat and connective tissue may be initiated during glutaraldehyde treatment and continued during the subsequent saline rinses, taking care to avoid damage to the pericardium. After completion of mechanical cleaning, which requires approximately 15 min, the pericardium may be released from the fixation surface to permit more thorough rinsing in physiological saline. The rationale for the chosen glutaraldehyde protocol is discussed below.

### 2.2. Cusp Preparation

Following preparation, the pericardium is carefully inspected, and any areas of mechanical damage are identified and marked (Figure 1B). Three valve cusps of size 4 are outlined using the Fehling template (AV-cusp gauge, tricuspid, size 4, item MVC-4; Fehling Instruments, Karlstein, Germany), ensuring that all damaged areas are avoided. Whenever sufficient intact tissue is available, an additional fourth cusp is outlined as a reserve in the event of intraoperative damage during suturing. The superior portion of each cusp is extended to form triangular “ears” measuring approximately 2.5 × 5 mm. The midpoint of the cusp base is marked, together with reference points located 5 mm from each superior commissural edge. These markings facilitate correct alignment and orientation during implantation (Figure 1B). The three cusps, including their corresponding triangular ears, are then excised.

### 2.3. Preparation and Marking of the Bovine Pericardial Patch

**Figure 2:**
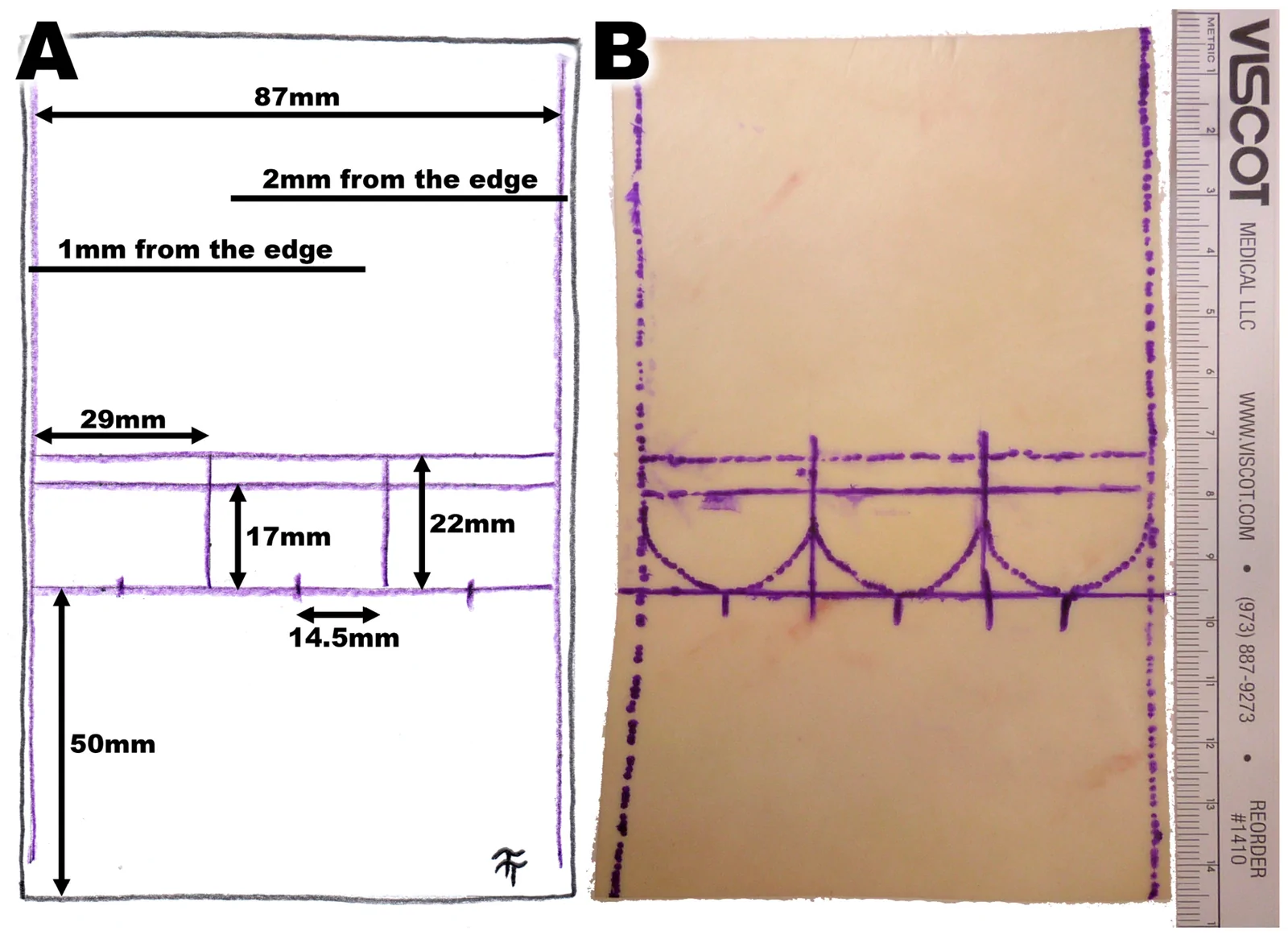
Layout of suture lines on a bovine pericardial patch (14 × 9 cm). (**A**) Geometric schematic demonstrating the course of the suture lines. (**B**) Photograph of the bovine pericardium with the planned suture lines outlined prior to suturing.

A bovine pericardial patch measuring 14 × 9 cm (SJM™ Pericardial Patch with EnCap Technology; Ref. C0914; manufactured by Glycar S.A. (Pty) Ltd., Irene, South Africa; distributed by St. Jude Medical, Inc., St. Paul, MN, USA, now part of Abbott, Abbott Park, IL, USA) is used for conduit construction. According to the manufacturer’s instructions for use, this patch is prepared from bovine pericardium cross-linked with glutaraldehyde, with formaldehyde used during sterilization and a subsequent proprietary (EnCap) treatment intended to decrease residual aldehyde activity in the tanned tissue; for this patch size, the manufacturer specifies a thickness of 0.2–0.4 mm. The device is CE-marked (Council Directive 93/42/EEC; notified body 0124), and US federal law restricts its sale to, or on the order of, a physician. It is supplied sterile in a container with a 2% propylene-oxide-derived preservative solution (converted to 2% propylene glycol before shipment) and is stored at room temperature or under refrigeration, but not frozen, until use; immediately before use it is removed from the container with sterile atraumatic forceps and briefly soaked in a bath of 500 mL sterile physiological saline, after which it is ready for use. The patch is intended for single use and is not re-sterilized or reused. It is then dried, and the planned suture lines are drawn on its mesothelial surface. Two tubularization lines are marked on the two opposite long (14-cm) edges of the patch, 1 mm from one edge and 2 mm from the other. This slight asymmetry is intended to improve hemostatic sealing after tubularization. After subtraction of these margins, the usable width is 87 mm, which after tubularization corresponds to an internal circumference of approximately 8.7 cm and an internal diameter of approximately 27.7 mm. The cusp attachment baseline is drawn parallel to the short edge, approximately 5 cm from it. A second line, corresponding to the termination of the running cusp suture, is marked 17 mm above the attachment baseline. The line used for fixation of the superior portions of the cusps is drawn an additional 5 mm higher, corresponding to a total distance of 22 mm from the cusp attachment baseline. The resulting cusp implantation field measures 87 × 22 mm and is divided into three equal segments, each measuring approximately 29 × 22 mm and corresponding to one cusp. The midpoint of each segment is marked along the cusp attachment baseline (Figure 2A). From the midpoint of each segment, two quarter-circular arcs, each with a radius of 14.5 mm, are drawn toward the adjacent intercusp boundary lines or tubularization lines. Together, these arcs define a semicircular cusp attachment line with a width of 29 mm (Figure 2B). In principle, the layout scales proportionally: the cusp segment width equals π × the target internal diameter divided by three, and the arc radius equals half of the segment width; this adaptation has not been tested and should be regarded as a geometric hypothesis rather than a validated sizing algorithm. Patient-specific sizing was not required in the four cases reported here, in which the same approximately 27.7-mm conduit was used in all patients. A printable template at 1:1 scale is provided as Supplementary Materials.

### 2.4. Cusp Implantation

**Figure 3:**
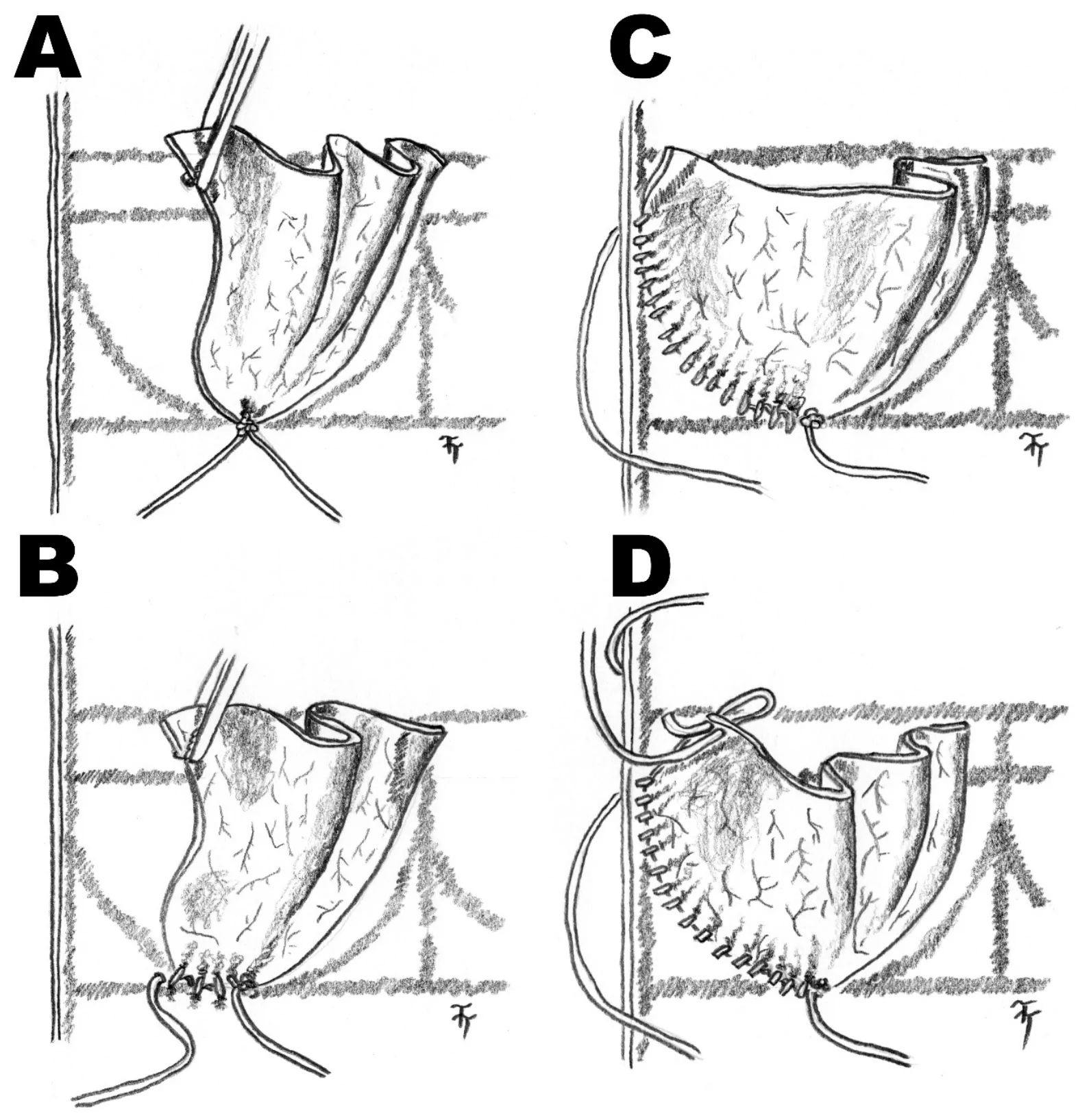
Schematic illustration of suturing one half of a single cusp to the bovine pericardial patch. (**A**) Initial fixation of the midpoint of the cusp fashioned from autologous pericardium to the corresponding midpoint marked on the bovine pericardial patch. (**B**) Placement of the first three stitches in an asymmetric 1:3 ratio, with shorter spacing between bites on the bovine pericardial patch and wider spacing between bites on the autologous pericardial cusp. (**C**) Appearance after completion of the running suture continued toward the commissural direction up to a height of 17 mm from the base of the future cusp, with subsequent stitches placed in a 1:1 ratio. After completion, the suture exits on the external surface of the bovine pericardial patch. (**D**) Placement of an additional stitch at the superior edge through the triangular “ear” of the autologous pericardial cusp. The limb passing through the lateral margin of the ear exits through the bovine pericardium to its external surface, whereas the limb passing through the junction of the ear and the cusp body remains temporarily free on the internal side and will be used for the corresponding stitch at the opposite end of the three-cusp assembly.

For improved handling, the bovine pericardial patch is secured to a circular suture organizer using four sutures, for example, a Crawford Neo Suture Holding Ring (detachable, diameter 250 mm, item 03-8000; Geister Medizintechnik GmbH, Tuttlingen, Germany). Each cusp is individually sutured to the smoother mesothelial surface of the bovine pericardial patch using 5-0 monofilament polypropylene suture (Surgipro™, Covidien/Medtronic, Mansfield, MA, USA). The cusp is oriented with its mesothelial surface facing the mesothelial surface of the bovine pericardium. This configuration is intended to minimize the thrombogenicity of the newly formed valve sinuses. For descriptive purposes, the mesothelial surface of the cusp is referred to as the arterial (outflow) side, whereas the non-mesothelial surface is referred to as the ventricular (inflow) side, because it will ultimately face the ventricular cavity during diastole. Suturing is initiated by fixing the midpoint of the cusp base to the corresponding midpoint of the cusp attachment baseline within the designated cusp field (Figure 3A). The first three stitches are placed asymmetrically in a 1:3 ratio, with larger bites taken through the autologous cusp and smaller bites through the bovine pericardium (Figure 3B); this is intended to accommodate the difference between the length of the cusp attachment edge and that of the curved suture path without puckering. The running suture is then continued symmetrically along the quarter-circle to a height of 17 mm above the cusp attachment baseline. The suture is adjusted so that exactly 5 mm remains between the final needle exit point and the superior edge of the cusp, as indicated by the previously placed reference marking. The needle is then passed through the bovine pericardial patch and exits on its non-mesothelial surface (Figure 3C). The same technique is applied to both halves of each cusp. The corresponding pairs of suture ends on the non-mesothelial surface of the bovine pericardium are tied without excessive tension to avoid puckering of the cusp edge. Sutures exiting along the opposing tubularization lines are left untied until completion of conduit tubularization.

### 2.5. Commissural Suturing

**Figure 4:**
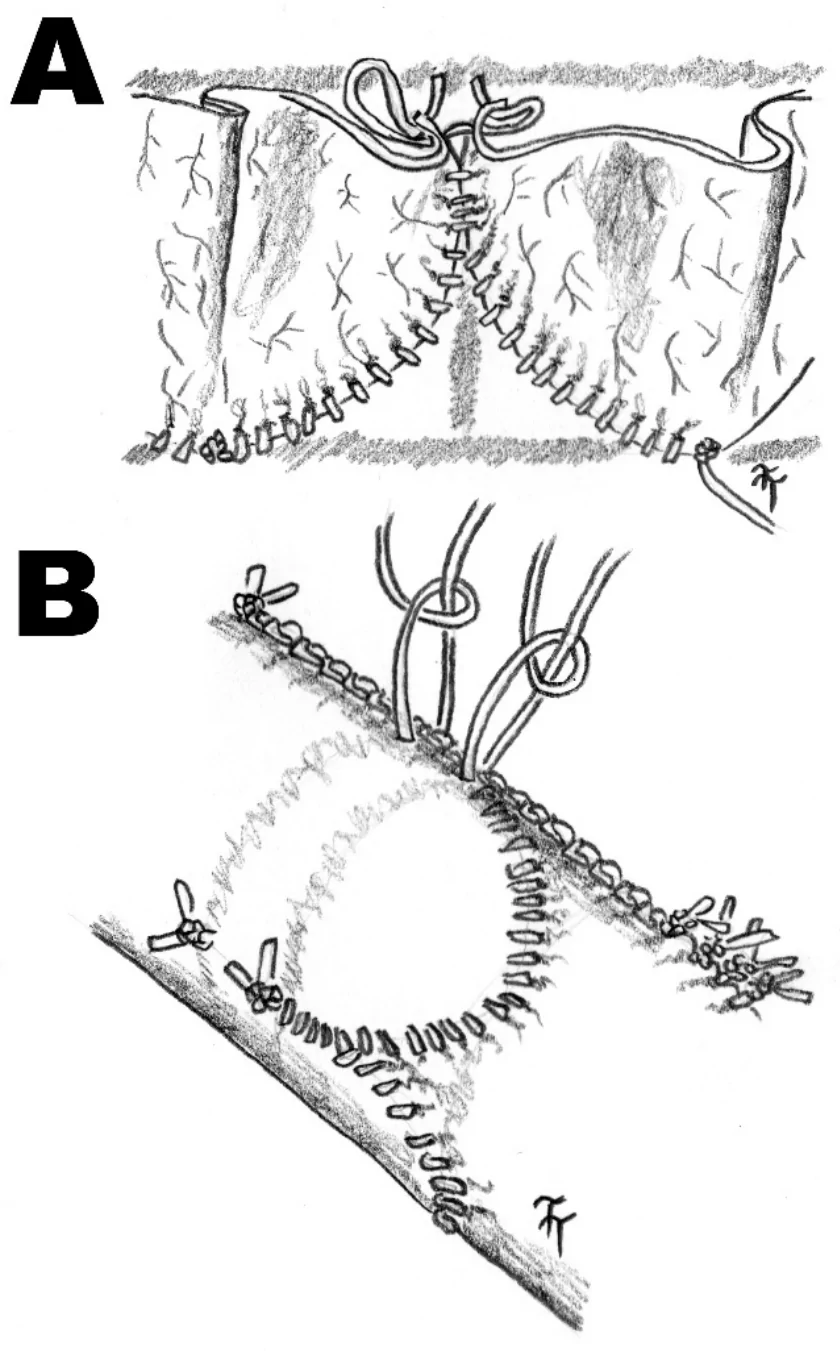
Schematic illustration of the final suture configuration. (**A**) Commissural suture connecting the “ears” of adjacent cusps. The suture passes from the external surface of the bovine pericardium to the internal surface at a height of 22 mm from the cusp base, then sequentially through the margin of the left cusp ear (ventricular to arterial side), the junction of the ear and the body of the left cusp (arterial to ventricular side), the junction of the ear and the body of the right cusp (ventricular to arterial side), and the margin of the right cusp ear (arterial to ventricular side). The suture then passes again through the bovine pericardium at the same height (22 mm from the cusp base) to the external surface, where it is tied. (**B**) Final knotting of the running suture together with the commissural suture passing through the cusp ears of the marginal cusps, after tubularization of the bovine pericardial patch.

The free superior margin of each cusp ear is secured to the bovine pericardium at a level 22 mm above the cusp attachment baseline, corresponding to the commissural region (Figure 3D). The commissural suture is passed from the non-mesothelial to the mesothelial surface of the bovine pericardium. It is then passed through the margin of the first cusp ear from the ventricular to the arterial side, followed by passage through the junction between the ear and cusp body from the arterial to the ventricular side. The suture is subsequently passed through the adjacent cusp in the reverse direction and returned through the bovine pericardium to its non-mesothelial surface, where it is tied (Figure 4A). The same technique is applied to the cusp ears located along the tubularization lines. However, these sutures are left untightened until tubularization has been completed (Figure 4B). During tightening of each commissural suture, the ventricular surfaces of the adjacent cusp ears are maintained in close apposition to the bovine pericardium to ensure proper commissural alignment and cusp coaptation.

### 2.6. Tubularization and Valve Competence Testing

**Figure 5:**
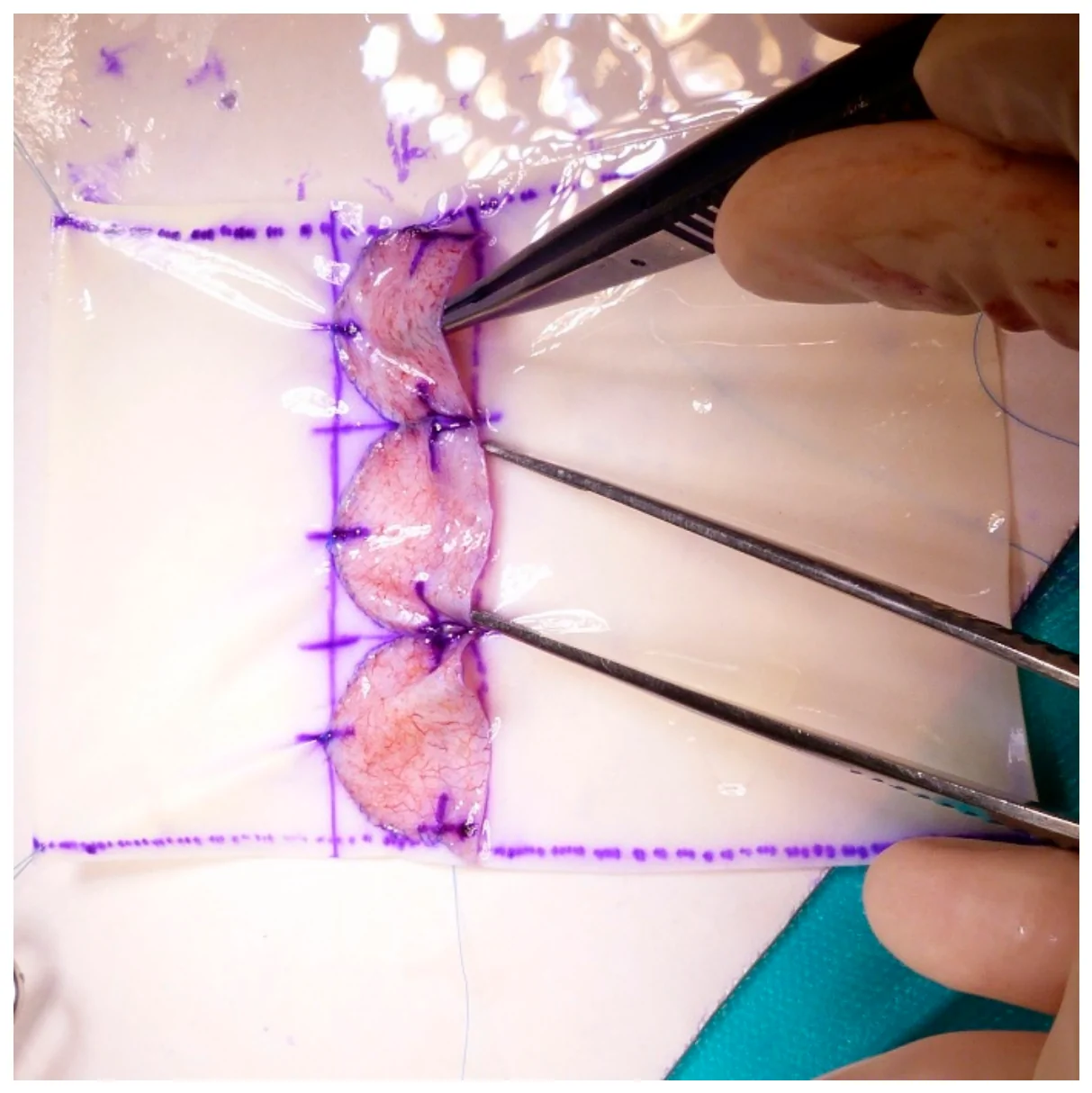
Appearance after suturing all three cusps to the bovine pericardial patch, before tubularization of the pericardial patch.

**Figure 6:**
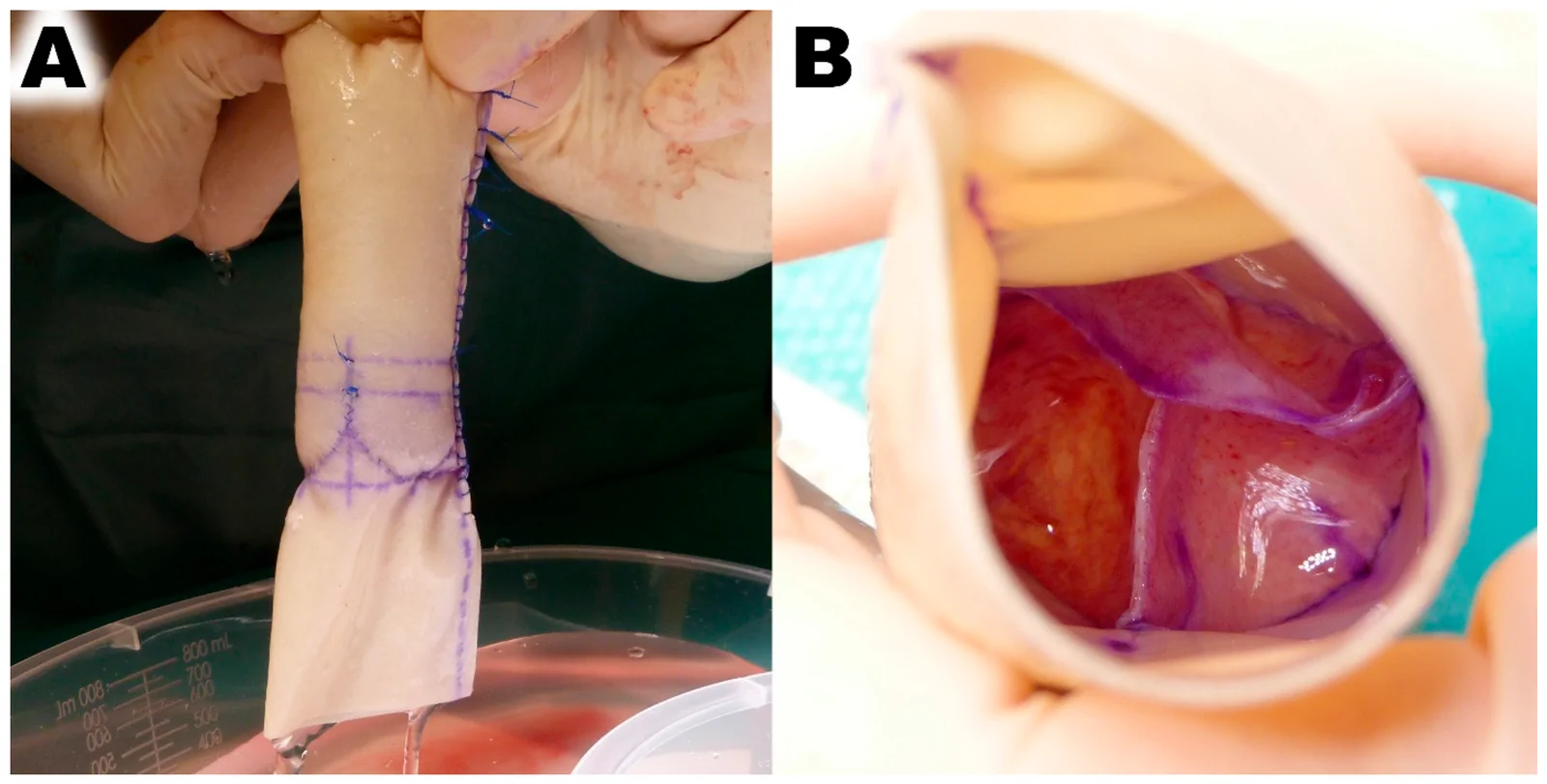
Final appearance of the prepared conduit. (**A**) Saline testing of the newly constructed valve demonstrating valve competence. (**B**) View of the neo-valve from the arterial side showing adequate coaptation of the neo-cusps.

After all three cusps have been implanted (Figure 5), the bovine pericardial patch is tubularized along the premarked lines located 1 and 2 mm from its long edge, using the same 5-0 monofilament polypropylene suture (Surgipro™, Covidien/Medtronic, Mansfield, MA, USA) used for cusp implantation and commissural fixation, described above. In portions of the conduit where no subsequent length adjustment is anticipated, a continuous over-and-over suture may be used. Interrupted sutures are recommended in regions where subsequent modification of conduit length may be required during implantation. After tubularization, the adjacent running sutures of the two marginal cusps and the commissural sutures located along the tubularization line are tied (Figure 4B). Valve competence is assessed by saline testing: saline is poured into the valve, and the cusps must retain the fluid level. This is followed by an orientative test in which saline is injected manually with a syringe to exclude a significant leak at higher pressure; pressure is not measured. Cusp coaptation and mobility are assessed visually (Figure 6): coaptation is considered adequate when the free edges of all three cusps appose each other along their full length by naked-eye inspection, as illustrated in Figure 6B, and mobility is considered adequate when no cusp has been inadvertently captured by the tubularization suture, allowing unrestricted excursion of all three cusps throughout the saline test. The valve is considered satisfactory when all three cusps are freely mobile throughout their full range and no jet is visible to the naked eye during the syringe test; minor, diffuse leakage around the suture line is considered acceptable. If a leak is present, its etiology is evaluated: a paravalvular leak is corrected with an additional stitch, and a central jet caused by cusp prolapse is corrected by central plication of the cusp. The completed conduit should be implanted without unnecessary delay. Until implantation, it is maintained in physiological saline (0.9% NaCl). The conduit is prepared with a length of up to 14 cm and is trimmed by the surgeon during implantation according to the patient’s anatomy; the 22-mm valve-bearing segment must not be shortened. To minimize the risk of conduit compression or kinking, the conduit is routed without redundant length and is secured without tension at both anastomoses.

## 3. Clinical Application

The conduit described above was used in four patients undergoing a modified Ross procedure at our institution between May and December 2025; no other modified Ross procedure was performed during this period, so the four cases were consecutive within this indication. Patients were eligible if they had an indication for aortic valve replacement, were considered anatomically suitable candidates for a pulmonary autograft (Ross) procedure, and declined a mechanical prosthesis; a homograft-based right ventricular outflow tract reconstruction was not offered because pulmonary homografts are unavailable at our institution. All patients provided written informed consent after being informed of the non-standard nature of the conduit and of the alternatives. Clinical, operative, and echocardiographic data were retrospectively extracted from the institutional electronic medical record for the index hospitalization and, where available, the first outpatient assessment within 30 days of surgery; this dataset and its ethics approval are described under Institutional Review Board Statement. The predefined outcomes for this report were: technical feasibility of conduit construction and implantation; early (in-hospital) echocardiographic function of the neoconduit and of the pulmonary autograft; and early postoperative adverse events, defined as death, low cardiac output syndrome, right ventricular dysfunction, clinically significant arrhythmia, bleeding requiring reoperation or transfusion, infection, thromboembolism, renal complications, neurological events, mechanical ventilation beyond 24 h, and unplanned readmission within 30 days. Baseline and operative characteristics are summarized in Table 1, and early outcomes in Table 2. Longer-term outcomes were intentionally not analyzed for this report and are addressed separately in the Discussion and Limitations.

Construction of the conduit was started by the surgical team that harvested the pericardium and was continued in parallel with the main operation in an adjacent operating room, requiring approximately 60 min; it therefore did not prolong the aortic cross-clamp time. The same steps can be performed on a side table when the size of the operating room allows. The same standardized neoconduit geometry (internal diameter approximately 27.7 mm) was used in all four patients, and no reduction in the conduit diameter was necessary. The neoconduit was anastomosed distally with a 5-0 suture at the junction of the pulmonary trunk and its bifurcation and proximally to the right ventricular outflow tract with a 4-0 suture, with the proximal end trimmed obliquely as required; the exact trimmed conduit length delivered to each patient was not separately recorded beyond the standardized pre-tubularization length of up to 14 cm stated above. Postoperatively, patients received aspirin for three months, corresponding to the regimen used after bioprosthetic aortic valve replacement, together with anti-inflammatory therapy (ibuprofen 3 × 400 mg daily for 3–6 months), recommended by analogy with practice after pulmonary homograft implantation with the aim of prolonging conduit durability [3].

In Patient 2, reoperation on the first postoperative day was prompted by progression of pulmonary autograft regurgitation in the aortic position. At the index procedure, intraoperative transesophageal echocardiography demonstrated grade I–II residual autograft regurgitation, whereas the neoconduit in the pulmonary position showed only trace regurgitation, with preserved biventricular function. Repeat transesophageal echocardiography on the first postoperative day demonstrated progression of autograft regurgitation to moderate-to-severe (grade II–III). At reoperation, direct inspection revealed pliable and structurally unremarkable autograft cusps with complete coaptation on visual and saline testing; the regurgitation was attributed to the short geometric height (approximately 15 mm) of the noncoronary cusp rather than to an identifiable technical defect, and the autograft was replaced with a bioprosthesis. The neoconduit, which constituted an anatomically separate right ventricular outflow tract reconstruction, was not surgically explored or manipulated during the reoperation. Subsequent postoperative echocardiographic assessment demonstrated preserved neoconduit function, with unobstructed flow and trace regurgitation before hospital discharge and at the 30-day follow-up (Table 2). Taken together, the localization and progression of regurgitation to the pulmonary autograft, the anatomical findings at reoperation, and the preserved postoperative function of the neoconduit provided no evidence that conduit dysfunction contributed to the indication for reoperation. No clinical or echocardiographic evidence of conduit kinking, compression, or distortion was observed in any of the four patients.

Early transthoracic echocardiography demonstrated unobstructed flow and no more than trace-to-mild regurgitation across the neoconduit in the pulmonary position in all four patients (Table 2). One patient (Patient 2) required reoperation on the first postoperative day for moderate-to-severe regurgitation of the pulmonary autograft in the aortic position, as described above; postoperative echocardiography subsequently confirmed preserved neoconduit function. Other early adverse events, listed individually in Table 2 and summarized by category in Table 3, included transfusion-requiring bleeding in two patients, transient atrial fibrillation in two patients, one episode of postoperative encephalopathy with a negative structural brain workup, and one 30-day readmission for a pleural effusion; no renal complications, no thromboembolic event, and no infection occurred in any patient. One patient (Patient 2) required mechanical ventilation beyond 24 h, in the context of the reoperation and transient encephalopathy described above, and was weaned to high-flow nasal oxygen by the second to third postoperative day; the other three patients were extubated within a few hours of surgery. A complete category-by-category summary of early adverse events is given in Table 3. Given the small, single-institution sample and the intentionally early cut-off applied to this report, these figures should not be interpreted as definitive safety estimates; they are reported in full, on a per-patient basis, to allow transparent assessment of the early clinical experience underlying this technical description. As stated in the Discussion and Limitations, this experience documents technical feasibility only; hemodynamic performance, structural durability, and safety beyond the early postoperative period remain to be established in larger studies with standardized, prospective follow-up.

## 4. Discussion

### 4.1. Ozaki Procedure and Pulmonary Valve Substitutes

The Ozaki procedure, in which aortic cusps are replaced by templated cusps fashioned from glutaraldehyde-treated autologous pericardium, was introduced in 2011 [4] and subsequently modified [5]; favorable mid-term results have been reported, particularly in selected younger patients [6,7]. Pulmonary valve replacement is complicated by the fact that most available substitutes, other than homografts, are designed for the aortic position [8]. Bioprosthetic valves are widely used, but reintervention rates increase with time, particularly in young patients [8,9,10]. Mechanical prostheses require anticoagulation, and early failures were largely attributed to inadequate anticoagulation [8,11]. Stentless xenografts are considered an alternative to homografts in the Ross procedure, but their expected durability is inferior [3]. Application of the Ozaki technique to the pulmonary valve has been described in isolated case reports [12,13,14]. To our knowledge, construction of a complete valved conduit from Ozaki-inspired autologous pericardial cusps for right ventricular outflow tract reconstruction has not been described beyond our initial clinical report [2].

### 4.2. Valve Neocuspidization Within Tubular Grafts

One of the first reports on valve neocuspidization within a tubular graft was published by Komarov et al. [15]. Individually calculated leaflets were sutured to the external surface of an inverted graft, which was then restored to its original configuration, or alternatively to the inner surface of a longitudinally opened graft that was subsequently reclosed. Pericardial conduits have also been used for reconstruction of the aortic root and ascending aorta in combination with mechanical or stented bioprosthetic valves [16].

### 4.3. Geometric Considerations

In the native aortic valve, the free margin of each cusp is longer than its geometric height, defined as the distance from the nadir of cusp insertion to the free edge [17]. Attempts to calculate the optimal dimensions of neocusps and neocommissures for reconstruction or de novo construction of the aortic valve date back to the beginning of the 21st century [15,18]. Komarov et al. defined the optimal free-margin length of a neocusp as one-half of the final tubular graft diameter multiplied by the square root of three [15], which for a graft with a final diameter of 28 mm gives approximately 24.2 mm. Tubularization of the bovine pericardial patch in our technique produces a conduit with an internal circumference of approximately 8.7 cm, corresponding to a diameter of approximately 2.77 cm. Because the cusp is curved and non-rectangular, its area cannot be reliably derived from its maximal width and height alone, and no planimetric measurement of cusp area was performed. The present geometric considerations are therefore based on linear dimensions and proportional relationships, and the construct should be regarded as a theoretical approximation rather than direct evidence of physiological equivalence.

### 4.4. Treatment of Autologous Pericardium

The use of autologous pericardium for valve reconstruction dates back to the 1960s [15]. Glutaraldehyde treatment of autologous pericardium was subsequently introduced for aortic valve reconstruction by Duran et al. in 1995 [15,19], and the approach became widely recognized through the work of Ozaki et al. [4,6]. Reported protocols for autologous pericardium use 0.6% glutaraldehyde for 6–10 min followed by saline rinses [12,14,15,20]. Both the concentration of glutaraldehyde and the duration of exposure influence the microstructure and mechanical properties of the treated pericardium: concentrations of 0.3125% and 0.625% with short exposure preserved the structural separation of collagen fibers, whereas 1.25% glutaraldehyde, or exposure approaching 1 h even at lower concentrations, was associated with collagen microstructural alterations [21]. The relationship with tissue elasticity is not necessarily linear [22], and cross-linking also depends on temperature [23]. This evidence derives from studies of human [21], bovine [22], and porcine [23] pericardium, which differ in structure and thickness and cannot be directly extrapolated to autologous human pericardium treated with the present protocol. In the present technique, the autologous pericardium was treated with 0.9% glutaraldehyde for 10 min at room temperature (approximately 22 °C), followed by two 5-min rinses in physiological saline. This concentration was chosen because the pericardium was easier to handle and retained its shape better than with 0.6% glutaraldehyde, whereas higher concentrations were avoided because they were associated with greater tissue damage in the literature [21]. The protocol is therefore a pragmatic modification of the 0.6% protocols and should not be regarded as equivalent to the established Ozaki procedure; its mechanical and microstructural effects have not been evaluated. The optimal concentration has not been established and would require a dedicated experimental study; long-term follow-up of our patients will show whether 0.9% is appropriate.

### 4.5. Autologous Versus Bovine Pericardium for the Cusps

Autologous pericardium was used for the cusps because we assume that this part of the conduit is the most mechanically stressed, and we extrapolate the results of the Ozaki procedure to the pulmonary position. Autologous pericardium briefly treated with glutaraldehyde is thinner and more pliable than commercial bovine pericardium, and the approach is economically advantageous because only one commercial patch is required. Harvesting takes only a few minutes, and cusp preparation runs in parallel with the main operation. Disadvantages are the need for pericardial harvesting and applicability limited to patients with sufficient intact pericardium. A direct comparison with conduits with bovine cusps has not been performed.

### 4.6. Limitations

This report describes a technique and has several limitations. Clinical experience is limited to four patients from a single institution with only early postoperative follow-up and demonstrates technical feasibility only; it does not allow conclusions regarding hemodynamic performance, safety, durability, or equivalence to pulmonary homografts. No ex vivo hydrodynamic, cyclic-loading, or mechanical testing of the construct was performed. The glutaraldehyde protocol has not been validated: residual glutaraldehyde may exert cytotoxic effects even after rinsing [24], and glutaraldehyde-treated tissue is prone to calcification, which is initiated mainly in devitalized cells [25]. This may be particularly relevant in the young patients in whom the Ross procedure is usually performed and in whom a long lifetime of the conduit is expected; degeneration, retraction, or loss of cusp mobility cannot be excluded. Sterility of the construct depends on strict aseptic handling during preparation, and pericardial thickness and quality vary between patients. Favorable outcomes of Ozaki reconstruction in the systemic circulation should be regarded as hypothesis-generating, since lower pressure in the pulmonary position does not eliminate the risk of calcification, retraction, thrombosis, infection, or conduit degeneration. The anti-inflammatory regimen was extrapolated from limited evidence on pulmonary homografts [3]. Finally, the effect of a larger residual pericardial defect on adhesions and re-entry has not been evaluated.

## 5. Conclusions

The described technique enables construction of a valved conduit from autologous and bovine pericardium using materials generally available in cardiac surgical practice and may represent an option for right ventricular outflow tract reconstruction when pulmonary homografts are unavailable. The present experience demonstrates technical feasibility only. Prospective evaluation with standardized follow-up is required to assess hemodynamic performance, structural degeneration, calcification, conduit stenosis or regurgitation, and freedom from reintervention before its safety, durability, and clinical role can be established.

## Figures and Tables

**Table 1:** Baseline and operative characteristics (CPB, cardiopulmonary bypass; neoSTJ, neo-sinotubular junction).

Patient	Age	Valve Pathology/Indication	Annulus Stabilization	NeoSTJ Graft	CPB Time (Minutes)	Cross- Clamp Time (Minutes)	Intraoperative Course
Patient 1 (woman)	33	Congenital bicuspid aortic valve; combined aortic severe stenosis with mild regurgitation	Not required	Vascular prosthesis No. 22	220	195	Uneventful
Patient 2 (man)	47	Bicuspid aortic valve; severe aortic stenosis with ascending aortic aneurysm (55 × 53 mm)	Ring No. 28	Vascular prosthesis, upsized intraoperatively to No. 32	323	204 + 58	Bleeding; re-clamping and revision of the right coronary-artery button anastomosis
Patient 3 (man)	44	Bicuspid aortic valve; combined aortic stenosis and regurgitation	Not required	Vascular prosthesis No. 26	194	168	Uneventful
Patient 4 (man)	32	Bicuspid aortic valve; severe aortic stenosis with mild regurgitation	Ring No. 34	Vascular prosthesis No. 24	255	217	Prolonged procedure with bleeding

**Table 2:** Early outcomes (AST/ALT, aspartate aminotransferase/alanine aminotransferase; ICU, intensive care unit).

Patient	ICU Stay	Early Neoconduit Function	Early Autograft Function	Early Adverse Events	Reoperation	Discharge Status
Patient 1	4 days	No regurgitation; unobstructed flow	Mild regurgitation	Transient biventricular systolic dysfunction, treated with levosimendan and creatine phosphate; bilateral pleural effusion, managed conservatively; postoperative anemia	None	Discharged in stable condition
Patient 2	Prolonged	Trace regurgitation; unobstructed	Moderate-to-severe regurgitation with reoperation	Major bleeding requiring transfusion and coagulation-factor concentrates; paroxysmal atrial fibrillation; readmission at 30 days for a symptomatic left pleural effusion requiring drainage	Autograft valve replaced with a bioprosthesis; the neoconduit was not implicated and was left in place	Discharged in stable condition
Patient 3	4 days	Trivial regurgitation; unobstructed	Trace, intermittent regurgitation	One paroxysm of atrial fibrillation, no recurrence; moderate anemia requiring transfusion; left pleural effusion 55–60 mm, managed conservatively; transient AST/ALT elevation, resolved	None	Discharged in stable condition
Patient 4	early extubation; uncomplicated	Trivial eccentric jet; unobstructed	Mild regurgitation	Intraoperative bleeding requiring coagulation-factor concentrates; transient low-dose inotropic/vasopressor support; no arrhythmia (predischarge Holter: sinus rhythm, rare ectopy)	None	Discharged in stable condition

**Table 3:** Early adverse events by category, per patient (CT, computed tomography; LVEF, left ventricular ejection fraction).

Adverse-Event Category	Patient 1	Patient 2	Patient 3	Patient 4
Mortality	None	None	None	None
Low cardiac output syndrome	Yes—transient biventricular dysfunction	Yes—combined vasopressor/inotropic support; weaned by postoperative day 2	None	Yes—transient low-dose inotropic support; weaned early
Right ventricular dysfunction	Yes—transient, concurrent with the left-ventricular “stunning” above; resolved	Not specifically documented beyond overall LVEF	None	None
Arrhythmia	None	paroxysmal atrial fibrillation	paroxysm of atrial fibrillation	None
Bleeding	None requiring transfusion or reoperation	major bleeding; transfusion and coagulation-factor concentrates	Mild—postoperative anemia requiring red-cell transfusion; no reoperation	significant intraoperative bleeding; replaced with coagulation-factor concentrates; no reoperation for bleeding
Infection	None documented	None documented	None documented	None documented
Thrombosis/thromboembolism	None	None	None	None
Renal complications	None	None	None	None
Neurological events	None	transient postoperative encephalopathy; brain CT negative for acute hemorrhage or ischemia	None	None
Prolonged mechanical ventilation	None—extubated within a few hours	weaning attempt; transitioned to high-flow nasal oxygen by day 2–3; exact	None	None
Unplanned readmission (≤30 days)	None	readmitted at the 30-day follow-up visit for a symptomatic left pleural effusion requiring drainage	None	None

## Data Availability

The data presented in this study are available within the article. Additional de-identified data may be available from the corresponding author upon reasonable request, subject to institutional and privacy restrictions.

## Supplementary Materials

The following supporting information can be downloaded at https://www.mdpi.com/article/10.3390/jcm15197684/s1. Printable template of the bovine pericardial patch at 1:1 scale (print at 100%).
