# Supplementary material for: Autologous Pericardial Cusps Within a Tubularized Bovine Pericardial Conduit for Right Ventricular Outflow Tract Reconstruction During the Modified Ross Procedure: A Technical Note
Source: J Clin Med. 2026 Oct 4;15(19):7684. doi: 10.3390/jcm15197684 (PMC13666063; doi:10.3390/jcm15197684)
Supplement: Supplementary file 1 [file jcm-15-07684-s001.zip › jcm-4498328-supplementary.pdf]

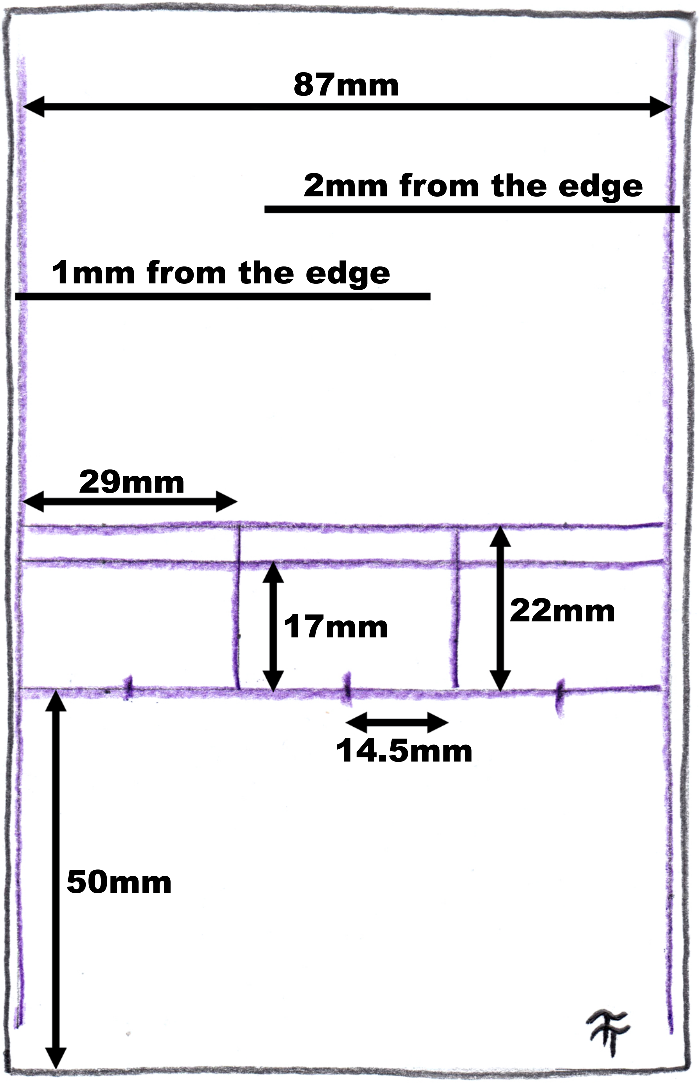
87mm
2mm from the edge
1mm from the edge
29mm
17mm
22mm
14.5mm
50mm
